# Effectiveness of Psychotherapy on Suicidal Risk: A Systematic Review of Observational Studies

**DOI:** 10.3389/fpsyg.2019.00277

**Published:** 2019-02-19

**Authors:** Pablo Méndez-Bustos, Raffaella Calati, Francisca Rubio-Ramírez, Emilie Olié, Philippe Courtet, Jorge Lopez-Castroman

**Affiliations:** ^1^Department of Psychology, Universidad Católica del Maule, Talca, Chile; ^2^INSERM, University of Montpellier, Neuropsychiatry: Epidemiological and Clinical Research, Montpellier, France; ^3^Department of Psychiatry, Mount Sinai Beth Israel, New York, NY, United States; ^4^FondaMental Foundation, Créteil, France; ^5^Department of Emergency Psychiatry and Post-Acute Care, Lapeyronie Hospital, CHU Montpellier, Montpellier, France; ^6^Department of Adult Psychiatry, Nîmes University Hospital, Nîmes, France

**Keywords:** effectiveness, psychotherapy, suicide attempt (SA), suicidal ideation (SI), systematic review

## Abstract

**Background:** Suicidal behavior is a major public health concern worldwide, and the interest in the development of novel and more efficient treatment strategies and therapies to reduce suicidal risk is increasing. Some recent studies have summarized the results of randomized clinical trials (RCTs) assessing the efficacy of psychotherapeutic tools designed to treat patients at suicidal risk. However, observational studies, which reflect real-world effectiveness and may use original approaches, have not been reviewed.

**Method:** The aim of this study is to systematically review the available scientific evidence issued from observational studies on the clinical effectiveness of psychotherapeutic tools designed to treat patients at suicide risk. We have thus performed a systematic search of PubMed and Web of Science databases.

**Results:** Out of 1578 papers, 40 original observational studies fulfilled our selection criteria. The most used psychotherapeutic treatments were dialectical behavioral therapy (DBT, 27.5%) and cognitive behavioral therapy (CBT, 15.0%) in patients with a diagnosis of borderline personality disorder (32.5%) and depression (15.0%). Despite the between-study heterogeneity, interventions lead to a reduction in suicidal outcomes, i.e., suicidal ideation (55.0%) and suicide attempts (37.5%). The content and reporting quality varied considerably between the studies.

**Conclusion:** DBT and CBT are the most widely used psychotherapeutic interventions and show promising results in existing observational studies. Some of the included studies provide innovative approaches. Group therapies and internet-based therapies, which are cost-effective methods, are promising treatments and would need further study.

## Introduction

Suicide is a global public health problem causing about one million deaths every year according to the World Health Organization (World Health Organization, 2018). Although the most relevant risk and protective factors associated with suicidal behavior have been identified (Table 1), the global suicide rates have remained relatively stable in the last years.

**Table 1:** Risk and protective factors associated with suicidal behavior.

**Risk factors**	**Protective factors**
**INDIVIDUAL-LEVEL**	
Prior suicide attempt(s)	Problem-solving skills
Mental disorders (Axis II diagnosis)	Frustration tolerance
Trauma or abuse history	Self-control
Hopelessness	Reasons for living and optimism
Stressful life events	Perceptions of positive health
Self-harm	Participation in sporting activities
Prior psychiatric hospitalization	–
Family history of suicide	–
Chronic illness and pain	–
Personality traits	–
Biomedical/physical determinants	–
**SOCIAL-LEVEL**	
Job or financial loss	Family relationships
Socio-economic disadvantage	Partnership
Relationship conflict, discord or loss	Social relationships and social support
Disaster, war and conflict	Religious or spiritual beliefs
Acculturation stress	Employment

*(McLean et al., 2008); (Ougrin et al., 2015); (World Health Organization, 2014)*.

One essential drawback for reducing suicide and suicide attempts is the lack of clear evidence on interventional programs directed to the population at risk (e.g., patient with suicide attempt history). Besides, knowledge about the efficacy of existing interventions is limited by the paucity of randomized clinical trials (RCTs) (Miller et al., 2017). Some interventions have shown to be efficacious, but the integration and dissemination of these programs in common clinical practice has proven to be an arduous task (Comtois and Linehan, 2006). The role of psychotherapy in suicide prevention is recognized but insufficient (Schneider, 2012). Current evidence supports especially the efficacy of Cognitive Behavioral Therapy (CBT) or Dialectical Behavioral Therapy (DBT), with a particular interest of problem-solving strategies (McMain et al., 2009; Rudge et al., 2017; Weinstein et al., 2017; Calati et al., 2018). Intensive outpatient support therapy, even if unspecific, is also a mainstay of suicide prevention guidelines (Mann et al., 2005; Zalsman et al., 2016). However, the evidence supporting these therapies and how to apply them is still scarce. Further research is needed to sustain existing results and design treatment plans contributing to a better treatment approach for the suicidal patient in different contexts, such as emergency room, primary care or inpatient units (Comtois and Linehan, 2006).

The creation of evidence-based guidelines for psychotherapy in suicide prevention is needed to improve the outcomes, especially in vulnerable groups presenting major social, psychiatric, or psychological risk factors (Valtonen et al., 2006; Rihmer, 2007; Fountoulakis et al., 2009; Rogers et al., 2018). Ideally, intervention strategies could follow a consensual methodology to ensure the coherence and comparability of results.

The aim of the current study is: (1) to systematically review observational studies exploring the effect of psychotherapeutic programs in the prevention of suicidal behaviors, (2) to describe the quality of this literature, (3) to identify innovative approaches, and (4) to propose recommendations for future observational research in this area. We planned to include only observational studies in order to assess literature that is not covered by recently published systematic reviews and meta-analyses (Sledge et al., 2014; Calati and Courtet, 2016; Hawton et al., 2016; Meerwijk et al., 2016; Krysinska et al., 2017; Leavey and Hawkins, 2017; Calati et al., 2018). Observational studies may help to assess the effectiveness of a psychotherapeutic strategy (Nallamothu et al., 2008), while RCTs are not necessarily representative of real-world situations because of their detailed inclusion and exclusion criteria (Faraoni and Schaefer, 2016). Thus, results from both RCTs (efficacy) and observational studies (effectiveness) provide valid evidence to improve clinical practice (Shadish et al., 2000; Berger et al., 2012).

## Materials and Methods

### Search Strategy

A systematic review was performed to identify the available published data on psychotherapeutic strategies addressing suicidal behavior. A broad free text search was made using the terms (psychotherap^*^ OR psychosoc^*^ OR psychologic^*^ OR acceptance and commitment therapy OR cognitive behavior^*^ therapy OR cognitive therapy OR dialectical behavior therapy OR dialectical behavior therapy OR interpersonal psychotherapy OR mentalization based treatment OR mindfulness OR problem solving therapy OR schema-focused therapy OR transference-focused psychotherapy) AND (effectiveness OR efficac^*^) AND (suicid^*^) for PubMed and Web of Science. Potentially relevant papers in all languages until March 2018 were accessed to review full texts. Additional articles were obtained through citation tracking of reviews/opinion articles and original papers. The titles, abstracts, and studies identified in the literature search were assessed by two reviewers (PMB and FRR). All studies matching the inclusion criteria were reviewed by the authors and disagreements were settled through discussion.

### Inclusion Criteria, Exposures, and Outcomes

**Figure 1:**
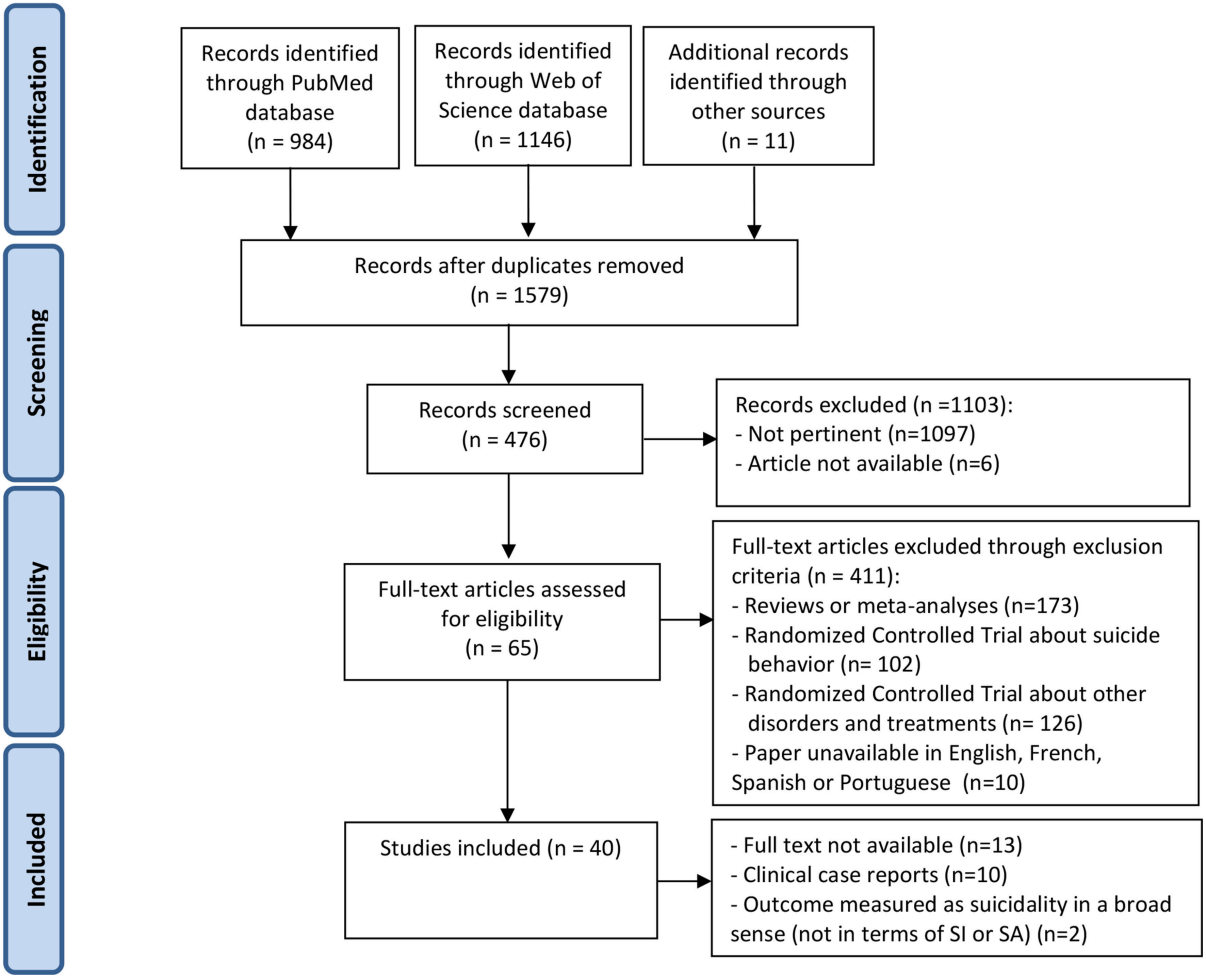
PRISMA flow diagram summarizing the systematic literature review and its results.

In this review we included only observational studies in populations presenting suicidal ideation, suicide plans, or suicide attempts and informing about the effect of a psychotherapeutic approach, either individual or group therapy, in terms of suicidal outcomes. Concerning suicidal outcomes we referred to established nomenclature (Turecki and Brent, 2015). In particular, suicidal ideation refers to thoughts about taking action to end one's life, while suicide attempt is a self-inflicted potentially injurious behaviour with a non-fatal outcome and with the intention to die (De Leo et al., 2006). Only papers in English, French, Spanish or Portuguese were included. A flow diagram summarizing the selection process can be found in Figure 1.

### Exclusion Criteria

All articles that focused on deliberate self-harm or non-suicidal self-injury, as opposed to suicidal behavior, were excluded. Systematic reviews, meta-analyses, randomized controlled trials and reports of clinical case studies were also excluded.

### Data Extraction and Quality Assessment

One independent reviewer (FRR) supervised by a senior reviewer (PMB) extracted the relevant data using a predesigned data extraction form. Disagreement between the two reviewers was solved by referring to two additional reviewers (JLC and RC). From each selected original observational study, the following data was extracted: sample size, gender, mean age, psychiatric diagnoses, psychotherapeutic strategy, duration, number of weekly sessions, follow up after therapy, assessment scales, main results, and methodological quality rating.

We assessed the methodological quality of papers using the Quality Assessment Tool for Observational Cohort and Cross-Sectional Studies created by the US National Heart, Lung, and Blood Institute (NHLBI) (NIH, 2018). This tool measures 14 different criteria which are then used to give each study an overall quality rating which is classified in good, fair, or poor. Two authors (PMB and FRR) applied this tool, they independently evaluated the items as “yes,” “no,” “not applicable,” “cannot determine” or “not reported.” This method was used to guide the quality rating of each study. In case of disagreement, consensus was reached through discussion. Based on previous articles (Koppen et al., 2016; Carbia et al., 2018) and our own assessment, we used the following threshold scores for the classification of the studies: good (>11), fair (6–9) and poor (<6).

## Results

### Description of the Studies

A total of 40 papers were identified as meeting the inclusion criteria (Figure 1). A detailed description of the studies can be found in Tables 2, 3. Studies focused on suicidal ideation (*n* = 23, 57.5%) or suicide attempts (*n* = 17, 42.5%) in adult or adolescent samples. A large majority reported a decrease either in suicidal ideation (22/23) or suicide attempts (15/17).

**Table 2:** Description of studies reporting the effects of psychotherapy on suicide ideation.

**Study**	**Sample size**	**Gender: (females: N, %)**	**Mean age**	**Psychiatric diagnoses**	**Psychotherapeutic strategy**	**Duration**	**Number of weekly sessions**	**Follow up after therapy**	**Assessment scales**	**Mains results (compared to baseline or other intervention)**	**NIH NHLBI quality rating**
Najavits et al., 1998	17	17, 100	35.9	PTSD/SD	CBT	3 months	Two	3 months	SBQ	Reduction of SI	Fair
Low et al., 2001	10	10, 100	28.7	BPD	DBT	12 months	One	6 months	BSSI	Reduction of SI	Poor
Katz et al., 2004	62	52, 83.8	15.4	NR	DBT v/s TAU	2 weeks (DBT)	Two	12 months	SIQ	Reductions of SI (both treatments)	Fair
Stanley et al., 2007	20	17, 85	32.2	BPD	DBT	6 months	NR	3 and 6 months	Self-report measures	Reduction of SI	Fair
Högberg and Hällström, 2008	14	11, 78.6	14.7	NR	Active multimodal psychotherapy	NR	Every 2 weeks	22 months	GAF	Reduction of SI	Fair
Miklowitz et al., 2009	22	NR	40.6	BD	MBCT	2 months	NR	NR	BSSI	Reduction of SI	Fair
Perepletchikova et al., 2011	11	6, 55	9.83	Depression Anxiety	DBT	1.5 months	Two	NR	MFQ	Reduction of SI	Poor
Diamond et al., 2012	10	8, 80	15.10	NR	ABFT	3 months	One	NR	SIQ	Reduction of SI	Fair
Ellis et al., 2012	20	16, 80	36.9	MD	CAMS Program	51 days	Two	NR	BSSI	Reduction of SI	Poor
Gutteling et al., 2012	34	34, 100	32.65	BPD	DBT Group Therapy	12 months	One (2 h)	NR	BDI and Dutch version of the SCL-90-R	Reduction of SI	Poor
Watts et al., 2012	299	166, 56	43	MDD	ICBT	1 week	Six online lessons	NR	PHQ-9	Reduction of SI	Fair
King et al., 2013	2070	1227, 63	33.2	D	Suicide prevention intervention (CBT-based).	2 months	Unlimited sessions (45–60 min. each session)	NR	MSSI	Reduction of SI	Poor
Petrakis and Joubert, 2013	65	26, 40	17 to 78	D	Assertive Brief Psychotherapy and Community Linkage	6 months	NR	6 months	BDI-II	Reduction of SI	Fair
Ward-Ciesielski, 2013	18	10, 56	40.19	NR	DBT	NR	One-time	1 month	SSI	Reduction of SI	Fair
Ducasse et al., 2014	35	15, 42.9	38.4	SBD	ACT	seven sessions	One	3 months	C-SSRS SSI score	Reduction of SI	Fair
Serpa et al., 2014	79	9, 11	60	NR	MBSR	2 months	One (2-h sessions)	NR	PHQ-9	Reduction of SI	Fair
Heisel et al., 2015	17	9, 53	70.1	MD	IPT	4 months	One (50–60 min. sessions for 16 weeks)	3 and 6 months	GSIS	Reduction of SI	Fair
Mewton and Andrews, 2015	484	232, 60.3	41.9	D	ICBT	1 week	Six online sessions	NR	PHQ-9	Reduction of SI	Poor
Rodzinski et al., 2015	680	473, 70	30.1	NB/PD	Intensive integrative psychotherapy with predominance of the psychodynamic approach	3 months	One session (individual) 10–15 sessions (group psychotherapy)	NR	Symptom Checklist KO“O”	Reduction of SI	Fair
Walser et al., 2015	981 Veterans	222, 22.6	50.5	D	ACT-D	12 months	One per month (total: 12–16 sessions)	NR	BDI-II	Reduction of SI	Fair
Teismann et al., 2016	105	70, 66.7	37.4	AD	Exposure-based treatment	Unlimited	30 sessions in total	NR	DSI-SS	No effects on SI	Fair
Weinstock et al., 2016	12	6, 50	47.3	BD	Adjunctive behavioral activation (BA)	5 months	One (16 sessions)	NR	MSSI	Reduction of SI	Fair
Flynn et al., 2017	71	61, 86	40	BPD	DBT	12 months	Two (individual and group therapy)	12 months	BSSI	Reduction of SI	Fair

*ABFT, Attachment-Based Family Therapy; ACT, Acceptance and Commitment Therapy; ACT-D, Acceptance and Commitment Therapy for Depression; AD, Anxiety Disorder; BD, Bipolar Depression; BDI, Beck Depression Inventory; BPD, Borderline Personality Disorder; BSSI, Beck Scale for Suicide Ideation; CAMS, Collaborative Assessment and Management of Suicidality; CBT, Cognitive Behavioral Therapy; C-SSRS, Columbia-Suicide Severity Rating Scale; D, Depression; DBT, Dialectical Behavior Therapy; DSI-SS, Depressive Symptom Inventory Suicidality Subscale; GAF, Global Assessment of Functioning; GSIS, Geriatric Suicide Ideation Scale; ICBT, Internet Cognitive Behavior Therapy; IPT, Interpersonal Psychotherapy; MBSR, Mindfulness-Based Stress Reduction; MBCT, Mindfulness-Based Cognitive Therapy; MD, Mood Disorder; MDD, Major Depressive Disorder; MFQ, Mood and Feeling Questionnaire; MSSI, Modified Scale for SI; NB, Neurotic Behavioral; NR, Not Reported; PD, Personality Disorders; PHQ-9, Patient Health Questionnaire; PTSD, Post-Traumatic Stress Disorder; SBQ, Suicidal Behaviors Questionnaire; SBD, Suicidal Behavior Disorder; SCL-90-R, Symptom Checklist 90–Revised; SD, Substance Dependence; SI, suicidal ideation; SIQ, Reynolds' SI Questionnaire-Jr.; SSI, Scale for SI; TAU, Treatment As Usual*.

**Table 3:** Description of studies reporting the effects of psychotherapy on suicide attempts.

**Study**	**Sample size**	**Gender: (females: N, %)**	**Mean age**	**Psychiatric diagnoses**	**Psychotherapeutic strategy**	**Duration**	**Number of weekly sessions**	**Follow up after therapy**	**Assessment scales**	**Mains results (compared to baseline or other intervention)**	**NIH NHLBI quality rating**
Hengeveld et al., 1996	9	9, 100	31	NR	CBT	2 months	One	10 months	BDI	No effects on repetition of SA	Poor
Clarkin et al., 2001	23	23, 100	32.7	BPD	Transference Focused Psychotherapy	12 months	Two	NR	PHI	Reduction in SA	Poor
Chiesa and Fonagy, 2003	40	31, 77.5	32.2	PD	Psychosocial community-based treatment v/s Long term residential treatment (hospital-based program)	12 months	Two	24 months	Structured interview	Reduction in SA (mainly in Psychosocial community-based treatment)	Fair
Chiesa et al., 2004	143	NR	32.8	PD	Psychoanalytically oriented residential specialist program v/s Phased “step-down” specialist psychosocial program v/s General community psychiatric model	12 months	Two	12 months	Structured interview	Reduction in SA (only in the step-down condition of specialist psychosocial program)	Fair
Jobes et al., 2005	55	19, 34	29.1	MD	CAMS v/s TAU	CAMS 7 sessions TAU 12 sessions	One	6 months	SSF	Reductions in SA (both treatments)	Poor
Hulbert and Thomas, 2007	27	27, 100	34	BPD	STP	6 months	NR	12 months	PHI	Reduction in SA	Poor
Petersen et al., 2008	66	56, 84.4	27.4	PD	Specialized short-term psychotherapeutic day treatment program v/s TAU	5 months	Three	6 months	Patients' self-reported suicidal acts	Reduction in SA (Specialized short-term psychotherapeutic day treatment program)	Fair
Stanley et al., 2009	110	84, 75.5	15.8	NR	Manualized cognitive behavioral treatment	6 months	One	NR	NR	Insufficient evidence	Poor
Fleischhaker et al., 2011	12	12, 100	13 to 19	BPD	DBT-A	4–6 months	NR	12 months	LPC	Reductions in SA	Poor
Andion et al., 2012	51	51, 100	25.63	BPD	Combined individual/group DBT v/s Individual DBT	12 months	One	18 months	Number of SA	Reduction in SA (both treatments)	Fair
Bales et al., 2012	45	32, 71.1	30.1	BPD	Manualized day hospital MBT	18 months	One	NR	SSHI	Reduction in SA	Fair
Alesiani et al., 2014	32	26, 81	44.41	BPD/PD	STEPPS Program	6–8 months	Two	12 months	Number of SA	Reduction in SA	Fair
Stiglmayr et al., 2014	47	43, 91.5	30.1	BPD	DBT	12 months	Two (individual and group therapy)	4 months	LPC	Reduction in SA	Fair
Fischer and Peterson, 2015	10	10, 100	16.20	BN	DBT	6 months	One or less (total: seven sessions)	6 months	BDI-II and Diary cards	Reduction in SA	Good
Kvarstein et al., 2015	64 (MBT) 281 (Ps. T.)	54, 84 (MBT) 233, 83 (Ps. T.)	26 (MBT) 30 (Ps. T.)	BPD	MBT v/s Psychodynamic treatment program	36 months	One (dynamic therapy group)	NR	Self-report questionnaire	Reduction in SA (MBT more effective)	Fair
Alonzo, 2016	22	11, 50	33.45	MD	PS–CCI	NR	3 months	3 months	NR	Reduction in SA	Poor
Boccalon et al., 2017	24	20, 83	41.0	BPD/PD	STEPPS program	5 months	One	6 months	Clinical interview	Reduction in SA	Fair

*BDI, Beck Depression Inventory; BN, Bulimia Nervosa; BPD, Borderline Personality Disorder; CAMS, Collaborative Assessment and Management of Suicidality; CBT, Cognitive Behavioral Therapy; DBT, Dialectical Behavior Therapy; DBT-A, Dialectical Behavioral Therapy for Adolescents; LPC, Lifetime Parasuicide Count; MBT, Mentalization-Based Treatment; MD, Mood Disorder; NR, Not Reported; PD, Personality Disorders; PHI, Parasuicidal History Interview; PHI, Parasuicide Harm Inventory; PS–CCI, Problem Solving, Comprehensive Contact Intervention; SA, suicide attempt; SSF, Suicide Status Form; SSHI, Suicide and Self Harm Inventory; STEPPS, Systems Training for Emotional Predictability and Problem Solving; STP, Spectrum group Treatment Programme; TAU, Treatment As Usual*.

The most frequently reported interventions consisted on DBT or CBT. The remaining interventions used strategies based on miscellaneous approaches such as interpersonal psychotherapy, psychodynamic oriented therapy and family therapy.

The studies focused on patients with the following mental disorders: borderline personality disorder (*n* = 13, 32.5%), depression (*n* = 6, 15%), mood disorders (*n* = 4, 10%), and personality disorders (*n* = 3, 7.5%). Only the study by Ducasse et al. (2014) considered suicidal behavior disorder, the diagnostic category proposed in DSM-5 (American Psychiatric Association, 2013).

Psychotherapies were heterogeneous in terms of their intensity (duration of intervention, number of sessions). The duration varied between 1 and 2 weeks (*n* = 3, 7.5%), 2–3 months (*n* = 8, 20%), 4–8 months (*n* = 11, 27.5%), 12–18 months (*n* = 10, 25%), and one that extended over a period of 36 months (2.5%). Three studies (7.5%) did not report the duration. Most interventions planned one (*n* = 16, 40%) or two sessions per week (*n* = 10, 25%). Follow-up after therapy was reported in 57.5% of the studies. Follow-up length varied from 1 month (*n* = 1, 2.5%), 3–6 months (*n* = 12, 30%), 10–18 months (*n* = 8, 20%) and 2–3 years (*n* = 2, 5%). Many studies compared only assessments before and after therapy (*n* = 1 7, 42.5%).

### Studies Focused on Suicide Ideation

Twenty-three studies were assessed. Results were positive overall, with a decrease of suicidal ideation rates in 95.7% of them. The most used psychotherapeutic treatments were DBT (*n* = 7, 30.4%) and CBT (*n* = 4, 17.4%). Interventions generally followed a weekly pattern (*n* = 12, 52.2%).

Two naturalistic interventions were focused on internet-based CBT to address suicidal ideation in depressed patients recruited by their primary care physician (Watts et al., 2012; Mewton and Andrews, 2015). The brief intervention consisted in six online sessions but the reported positive results, with a decrease in both suicidal ideation and depression levels from baseline, suggest the utility of this method in terms of cost and accessibility for the patients. Perepletchikova et al. (2011) applied an intensive version of DBT (2 weekly sessions during 6 weeks) to 11 children presenting mood symptoms and obtained good results: suicidal ideation and depressive symptoms decreased, while coping strategies improved. Heisel et al. (2015) performed a pilot study including 16 sessions of weekly interpersonal therapy for older adults. Compared to baseline assessments, suicidal ideation was lower at the end of the treatment and 6 months later. Petrakis and Joubert (2013) applied a brief assertive psychotherapy by social workers to 57 patients attending the emergency department. Patients, independently of their diagnosis, received a comprehensive evaluation, and linkage to community services was proactively encouraged to minimize drop-outs. In this program, psychosocial improvements were associated with a reduction in the level of depression and suicidal ideation, but the specifics of the therapy are not described. Another study by Högberg and Hällström (2008) used the active multimodal psychotherapy, an integrative approach combining different psychotherapeutic techniques in a case series of 14 suicidal adolescents. The approach comprised mood charting, psycho-education, well-being practice and trauma resolution (including eye movement desensitization and reprocessing).

Of note, two studies used exclusively group interventions and four combined both individual and group interventions. A very complete Irish program delivered individual and group DBT weekly sessions, as well as phone coaching and follow-up visits, for borderline personality disorder patients during 12 months (Flynn et al., 2017). Gutteling et al. (2012) also found that group DBT could be used to reduce suicidal ideation and depressive symptoms in borderline personality disorder. In general, group interventions were effective in reducing suicidal ideation and improving several other outcomes related to mental health.

### Studies Focused on Suicide Attempts

Seventeen studies examined the effect of interventions in reducing subsequent suicide attempts. Most of them provided positive results (*n* = 15, 88.2%), frequently using DBT methods (*n* = 4, 23.5%) and weekly sessions (*n* = 8, 47.0%).

The Collaborative Assessment and Management of Suicidality (CAMS) was compared to treatment as usual (TAU) in a small sample of suicidal outpatients (Jobes et al., 2005). A reduction in suicidality (including any suicidal behavior or suicidal thought) was found in both groups at the end of treatment but CAMS achieved similar results in a significantly lower number of sessions. Interestingly, CAMS was also associated with decreased medical health care utilization 6 months after the treatment.

Some psychosocial programs combined social interventions and psychoanalytic therapy (individual and group-focused) to improve the social functioning of patients with personality disorders. These programs included a step-down period of limited duration offering biweekly therapy in small groups, meetings with community nurses and psychiatric consultations. Participants were also encouraged to create social bounds in the community, which was considered by the authors as particularly useful in reducing the risk of suicide attempts (Chiesa and Fonagy, 2003; Chiesa et al., 2004).

Another program, named Systems Training for Emotional Predictability and Problem Solving (STEPPS) and combining CBT elements and skills training with a systems component, was found to reduce suicide attempts in personality disorders patients (Alesiani et al., 2014; Boccalon et al., 2017). STEPPS was also associated with better emotional regulation, fewer hospitalizations and suicide attempts 6 months after the end of the treatment (Boccalon et al., 2017). Finally, the delivery of a manualized problem-solving and comprehensive contact intervention (PS-CCI) to mood-disordered patients found a decrease in both suicidal ideation and suicide attempts 3 months later (Alonzo, 2016). The intervention was delivered in emergency settings and included an educative interview about problem-solving, the sending of a personalized postcard and a telephone call 3 months later.

### Quality Assessment

The large majority of the studies (*n* = 26) was qualified as “fair” at the quality assessment. Only one was scored “good” while 13 studies were considered “poor.” The most common caveats were the lack of sample size justification, not describing precisely the features of participants, showing weaknesses in the statistical methodology and making only pre- and post-test evaluations (with no further assessment).

## Discussion

Clinical decision-making regarding patients with suicidal risk is largely based on the experience of health care providers, rather than international guidelines. Suicidal patients are heterogeneous, and frequently non-adherent to treatment or follow-up. Since this variability is unlikely to be reflected in RCTs, we have tried to synthesize data from observational studies to complete the results of previous reviews and meta-analyses. The results seem to confirm the effectiveness of psychotherapeutic interventions for the management and reduction of suicidal risk. However, there is a lack of methodological consensus on how to apply these interventions, which limits the generalizability of the findings. In this domain, many observational studies, similarly to RCTs (Witt et al., 2018), do not offer detailed information about the components of psychotherapeutic interventions, such as the number of sessions, their frequency, the duration of follow-up, or the clinical features of the sample. Of note, some strategies, such as internet-based therapies, group therapy or community settings for the treatment, might prove particularly cost-effective.

According to our results, CBT and DBT appear to be the most used and effective psychotherapeutic interventions for patients presenting suicidal ideation or suicide attempts, even in the short-term. For instance, 1-week internet-based CBT (Watts et al., 2012; Mewton and Andrews, 2015) and a short 2-month CBT program to reduce suicide risk in primary care patients (King et al., 2013) were both effective in reducing suicidal ideation. Another short-term intervention (seven sessions) addressing suicidal behavior disorder with Acceptance and Commitment Therapy showed a decrease in both the frequency and intensity of suicidal ideation (Ducasse et al., 2014). Of note, no other study focused on the diagnostic category of suicidal behavior disorder, which is associated with the risk of attempting suicide in the short-term. To consider suicidal behavior as a trans-diagnostic entity could help to more accurately evaluate the effect of psychotherapeutic interventions.

However, the range of potential psychotherapeutic interventions for suicide prevention is not limited to CBT and DBT. Mindfulness-based strategies, integrative programs, CAMS, STEPPS, or PS-CCI, just to mention some, are promising possibilities. Besides, most studies were conducted in adults, but some interventions have shown promising results in extreme ages, such as DBT adapted for children (Perepletchikova et al., 2011) and interpersonal therapy for older adults (Heisel et al., 2015).

Luoma et al. (2002) found that ~45% of suicide victims had contact with primary care in the month prior to their death and 77% in the year before suicide. Since so far the evidence sustaining targeted psychotherapeutic interventions for patients at suicide risk is still scarce, a research effort to establish effective interventions is needed. Some interventions need to be tested in independent and larger samples to verify their utility before translation into common clinical practice could be considered (Glenn et al., 2015). Group CBT for the prevention of repeated suicide attempts is currently being compared to individual supportive therapy by our team in a multicenter randomized clinical trial (clinicaltrials.gov registration: NCT02664701). Indeed, the setting of the therapy (individual vs. group) does not appear to predict the outcome for several mental disorders (Pomini, 2004) and the group setting provides important pragmatic advantages, such as a more efficient use of human resources dedicated to patient care and subsequent cost savings.

According to the quality assessment, reviewed studies present frequent weaknesses at the methodological level. These deficits comprise mainly a restricted evaluation of the sample, a vague description of the intervention, the non-justification of the sample size, the lack of a blinded outcome assessment, and a limited time frame to examine the association between exposure and outcome. In addition, adjustment for relevant confounders, such as educational level, depression severity, or the concomitant use of psychotropic treatment, was not considered in the majority of the studies. A reassessment several weeks or months after the end of the psychotherapy is also needed to ascertain the duration of the effect. Importantly, the cost-effectiveness of psychotherapeutic programs, such as short programs and group psychotherapy, could be compared to pharmacological approaches or non-specific support therapy. Our review uncovers a high heterogeneity in type and intensity of psychotherapeutic programs to reduce suicidal behavior. A quite wide range of psychotherapeutic strategies may be efficacious to prevent suicidal behavior but the benefits of their application in real clinical conditions (effectiveness) is not yet clear. We also need to differentiate the specific effect of psychotherapy from the non-specific effect of any treatment implying intensive contact and follow-up with a physician, such as supportive therapy provided by a general practitioner. Stronger evidence regarding the specific aspects of psychotherapy that reduce the suicide risk is thus needed.

Both RCTs and observational studies provide relevant information for the interpretation of the efficacy and effectiveness of therapeutic strategies applied to different populations. Future observational studies in this area should provide precise measurements of the exposure, as well as a detailed description of the components of psychotherapeutic interventions and the outcome variables of interest. Additional recommendations include a consensual terminology, notifying patients of the potential risks of therapies during the informed consent process, and clear procedures for monitoring and reporting side effects (Guidi et al., 2018; Rozental et al., 2018).

In summary, further research is still needed to discern how to improve psychotherapeutic strategies in suicide prevention. Replication by independent groups of successful programs is particularly important to ensure generalizability of the findings (Miklowitz and Taylor, 2006; Glenn et al., 2015; Zalsman et al., 2016). Psychotherapeutic interventions seem to have a positive effect in patients with suicidal ideation and suicide attempts, but it is not yet possible to identify the most effective/efficacious psychotherapeutic approach. This is partly due to the very high number of interconnected factors that should be assessed, i.e., patient-clinician-treatment related factors. Artificial intelligence could be one further promising tool to answer to this complex question.

## Author Contributions

PM-B, JL-C, and RC conceived and designed the study and drafted the manuscript. PM-B and FR-R managed the literature searches and analyses. All authors revised the article critically and read and approved the final manuscript.

### Conflict of Interest Statement

The authors declare that the research was conducted in the absence of any commercial or financial relationships that could be construed as a potential conflict of interest.
